# Overcoming cellulose recalcitrance in woody biomass for the lignin-first biorefinery

**DOI:** 10.1186/s13068-019-1503-y

**Published:** 2019-06-29

**Authors:** Haibing Yang, Ximing Zhang, Hao Luo, Baoyuan Liu, Tânia M. Shiga, Xu Li, Jeong Im Kim, Peter Rubinelli, Jonathan C. Overton, Varun Subramanyam, Bruce R. Cooper, Huaping Mo, Mahdi M. Abu-Omar, Clint Chapple, Bryon S. Donohoe, Lee Makowski, Nathan S. Mosier, Maureen C. McCann, Nicholas C. Carpita, Richard Meilan

**Affiliations:** 1 Department of Biological Sciences, Purdue University, West Lafayette, IN 47907 USA; 2 Laboratory of Renewable Resource Engineering (LORRE), Department of Agricultural and Biological Engineering, Purdue University, West Lafayette, IN 47907 USA; 3 Department of Chemistry and Biochemistry, University of California, Santa Barbara, Santa Barbara, CA 93106 USA; 4 Department of Botany and Plant Pathology, Purdue University, West Lafayette, IN 47907 USA; 5 Department of Biochemistry, Purdue University, West Lafayette, IN 47907 USA; 6 Department of Forestry and Natural Resources, Purdue University, West Lafayette, IN 47907 USA; 7 Bindley Bioscience Center, Purdue University, West Lafayette, IN 47907 USA; 8 Department of Chemistry, Purdue University, West Lafayette, IN 47907 USA; 9 National Renewable Energy Laboratory, Biosciences Center, Golden, CO 80401 USA; 10 Department of Bioengineering, Northeastern University, Boston, MA 02115 USA; 11 Department of Chemistry and Chemical Biology, Northeastern University, Boston, MA 02115 USA; 12 Present Address: College of Biosystems Engineering and Food Science, Zhejiang University, 38 Zheda Rd, Xihu Qu, Hangzhou Shi, 310027 Zhejiang Sheng China; 13 Department of Food Science and Experimental Nutrition, University of São Paulo, Av. Prof. Lineu Prestes, 580, Bloco 14, São Paulo, SP 05508-000 Brazil; 14 Plants for Human Health Institute, North Carolina State University, 600 Laureate Way, Room 3227, Kannapolis, NC 28081 USA; 15 Department of Horticulture, University of Florida, 1253 Fifield Hall, P.O. Box 110690, Gainesville, FL 32611 USA; 16 Department of Food Science, University of Arkansas, Fayetteville, AR 72701 USA; 17 National Cancer Institute, National Institutes of Health, Bethesda, MD 20892 USA; 18 Purdue Center for Plant Biology, West Lafayette, USA

**Keywords:** Cellulose, Lignin, Recalcitrance, Catalysis, Delignification, Poplar

## Abstract

**Background:**

Low-temperature swelling of cotton linter cellulose and subsequent gelatinization in trifluoroacetic acid (TFA) greatly enhance rates of enzymatic digestion or maleic acid–AlCl_3_ catalyzed conversion to hydroxymethylfurfural (HMF) and levulinic acid (LA). However, lignin inhibits low-temperature swelling of TFA-treated intact wood particles from hybrid poplar (*Populus tremula* × *P. alba*) and results in greatly reduced yields of glucose or catalytic conversion compared to lignin-free cellulose. Previous studies have established that wood particles from transgenic lines of hybrid poplar with high syringyl (S) lignin content give greater glucose yields following enzymatic digestion.

**Results:**

Low-temperature (− 20 °C) treatment of S-lignin-rich poplar wood particles in TFA slightly increased yields of glucose from enzymatic digestions and HMF and LA from maleic acid–AlCl_3_ catalysis. Subsequent gelatinization at 55 °C resulted in over 80% digestion of cellulose in only 3 to 6 h with high-S-lignin wood, compared to 20–60% digestion in the wild-type poplar hybrid and transgenic lines high in guaiacyl lignin or 5-hydroxy-G lignin. Disassembly of lignin in woody particles by Ni/C catalytic systems improved yields of glucose by enzymatic digestion or catalytic conversion to HMF and LA. Although lignin was completely removed by Ni/C-catalyzed delignification (CDL) treatment, recalcitrance to enzymatic digestion of cellulose from the high-S lines was reduced compared to other lignin variants. However, cellulose still exhibited considerable recalcitrance to complete enzymatic digestion or catalytic conversion after complete delignification. Low-temperature swelling of the CDL-treated wood particles in TFA resulted in nearly complete enzymatic hydrolysis, regardless of original lignin composition.

**Conclusions:**

Genetic modification of lignin composition can enhance the portfolio of aromatic products obtained from lignocellulosic biomass while promoting disassembly into biofuel and bioproduct substrates. CDL enhances rates of enzymatic digestion and chemical conversion, but cellulose remains intrinsically recalcitrant. Cold TFA is sufficient to overcome this recalcitrance after CDL treatment. Our results inform a ‘no carbon left behind’ strategy to convert total woody biomass into lignin, cellulose, and hemicellulose value streams for the future biorefinery.

**Electronic supplementary material:**

The online version of this article (10.1186/s13068-019-1503-y) contains supplementary material, which is available to authorized users.

## Background

Lignin and the crystallinity of cellulose are two major recalcitrance factors impeding the biochemical or chemical conversion of the carbohydrates in lignocellulosic biomass to biofuels and bio-based products [1, 2]. Various physical, chemical, and biological pretreatments have been used to improve enzymatic yields of fermentable sugars from biomass, including dilute acids [3, 4], steam expansion in water [5, 6], ammonia fiber expansion (AFEX) [7, 8], and ligninolytic enzymes [9]. AFEX swells lignocellulosic biomass, enabling nearly complete enzymatic conversion of cellulose to glucose [5]. Other treatments, such as NaOH/urea or 85% phosphoric acid, solubilize cellulose for improved saccharification yield [10]. Ionic liquids (ILs), such as 1-butyl-3-methylimidazolium (BMIM) chloride, or cellulose solvents, such as *N*-methylmorpholine *N*-oxide (NMMO), solubilize cellulose at relatively low temperatures without inducing extensive modification [11–13], by dissolving lignin and reducing cellulose crystallinity from woody biomass [14–16]. Although production of ILs from lignin-rich residues promises to mitigate reagent costs [17], the recycling of such reagents together with recovery and separation of clean streams of desired reaction products remain energy-intensive processes in the context of biorefinery operations [18, 19].

Solubilizing cellulose in trifluoroacetic acid (TFA) is a promising alternative pretreatment [20], from which amorphous cellulose can be recovered as a gel following the addition of an alcohol [21]. A two-step distillation can be used to recover both the TFA and the alcohol for reagent recycling. The transition of crystalline cellulose into more soluble states in TFA produces highly amorphous cellulose upon rapid suspension in ethanol [22]. Low-temperature swelling of pure cellulose in TFA at − 20 °C caused minimal decomposition and was sufficient to increase the rates of hydrolysis by commercial enzyme cocktails or the chemical catalytic conversion to fuel precursors, such as levulinic acid (LA) and hydroxymethylfurfural (HMF) [22].

We report here that, in contrast to the behavior of cotton linter cellulose, lignocellulosic materials from poplar (*Populus* spp.) were resistant to low-temperature swelling in TFA and required subsequent heating to generate more amorphous forms of cellulose. Because lignin interactions with cellulose are considered a major source of recalcitrance in biochemical conversion pathways, we evaluated the effect of modified lignin composition on the swelling and gelatinization of cellulose in woody biomass particles. Monolignols (*p*-coumaryl alcohol, coniferyl alcohol, and sinapyl alcohol) are substrates for lignin synthesis and are polymerized by free-radical coupling into *p*-hydroxyphenyl (H), guaiacyl (G), and syringyl (S) subunits, respectively, within the lignin heteropolymer. The genes involved in the synthesis of the monolignol substrates for lignin biosynthesis are well characterized [23, 24]. We have generated populations of transgenic, fast-growing hybrid poplar (*Populus tremula* × *P. alba*, genotype INRA 717-1B4) trees with large differences in their content of G, 5-OH-G, and S units by overexpression of an Arabidopsis (*Arabidopsis thaliana*) *FERULATE 5*-*HYDROXYLASE* (*AtF5H*) gene, or downregulating native *F5H* and *CAFFEIC ACID O*-*METHYL TRANSFERASE* (*COMT*) genes using RNA interference (RNAi) constructs driven by native or constitutive promoters. The lignocellulosic materials from these poplar variants were resistant to swelling regardless of lignin composition. However, after heat-induced swelling in TFA at 55 °C, the high-S-lignin lines had significantly higher initial rates of digestion with an enzyme cocktail routinely used in saccharification assays and reached completion more rapidly compared to wild-type (WT), high-G, or high-5-OH-G lines.

We then evaluated the behavior of these lignin variants in TFA after the removal of lignin. Chemical catalytic conversion pathways that use lignin for synthesis of biofuels and bioproducts yield aromatic monomers as platform intermediates for transformation to hydrocarbons without decomposition of cellulose. For example, bimetallic Pd-Zn/C [25] and Ni/C [26] catalytic systems have been developed to depolymerize lignin via β-*O*-4 ether bond cleavage, and dimeric lignin model complexes and synthetic lignin polymers can be cleaved with near-quantitative conversions, solubilizing 80–90% of the total lignin. Almost one-half of the disassembled lignin from intact woody particles is recovered as two methoxy-substituted propylphenols [27]. Once lignin has been removed from biomass, tandem catalytic reactions designed for depolymerization and subsequent deoxygenation of cellulose and xylan to furans [28–30] can be used to convert the carbohydrate-enriched residues [26, 27]. We report here that catalytic delignification (CDL) using Ni/C doubles the rates of enzymatic hydrolysis of cellulosic residues of all poplar genetic variants, but higher initial rates of enzymatic digestion of the cellulosic residues were observed in the high-S-lignin lines. Nevertheless, a substantial amount of cellulose recalcitrance remains after CDL. Regardless of lignin composition, low-temperature swelling alone was substantial and sufficient for rapid and complete enzymatic hydrolysis and for maximal conversion of catalytically delignified materials to HMF and LA. These results have implications for both biorefinery operations and the composition of the biomass delivered to them.

## Results

### Generation of variation in lignin composition in transgenic poplar lines

Genetic constructs designed to increase the relative proportion of S-lignin by expressing an Arabidopsis *F5H* gene (*AtF5H1*), or to reduce S-lignin by suppressing expression of endogenous poplar *F5H* genes by RNA interference (RNAi)-mediated knockdown, were introduced into cell cultures of hybrid poplar by *Agrobacterium*-mediated transformation (Additional file 1: Table S1). Suppression of endogenous *COMT* expression by RNAi was designed to replace S-lignin units with 5-OH-G units, which are normally of low abundance in the WT poplar. A constitutive promoter from the Cauliflower Mosaic Virus (CaMV)* 35S* gene was used to drive expression in all cells, and a promoter sequence upstream of an Arabidopsis *CINNAMATE 4*-*HYDROXYLASE* (*AtC4H*) gene was used to target expression to lignifying cells. Transformed plants were regenerated by direct organogenesis, rooted on a selection medium, and acclimated in a greenhouse before field planting. Wood from oven-dried debarked stems of three-year-old trees was chipped and then milled through a 20-mesh screen to yield 1-mm particles.

**Fig. 1:**
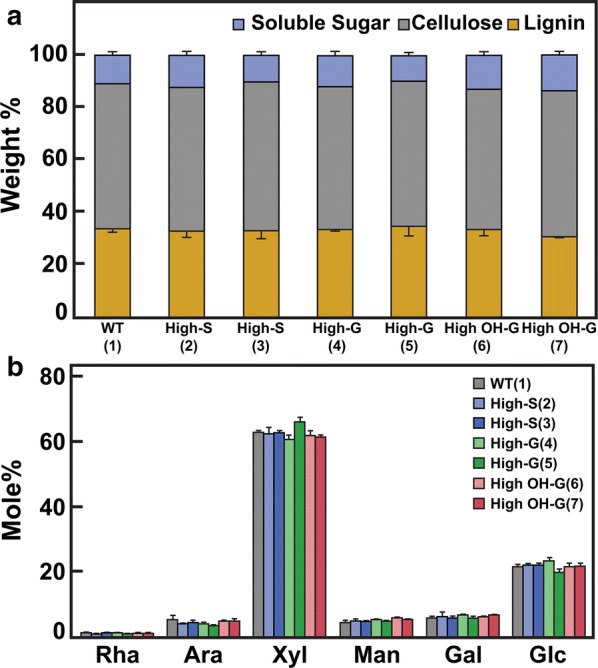
Composition of milled wood particles from wild-type (WT) and six transgenic poplar lines.** a** Mass balance of Klason lignin, cellulose, and non-cellulosic polysaccharides in wild-type and transgenic poplar lines.** b** Mole % of monosaccharides in 2 M TFA hydrolysates of the milled poplar wood. WT = (*Populus tremula* ×* P. alba* cv. INRA 717-1B4), high S-lignin =* pAtC4H:AtF5H* (OE) (2 lines); high G-lignin =* pAtC4H:PtF5H2* RNAi and* p35S:PtF5H2* RNAi, and high OH-G-lignin =* pAtC4H:PtCOMTa* RNAi and* p35S:PtCOMTa* RNAi. Values are the means ± S.D. of three determinations.

Wood particles from WT and transgenic trees comprised 34 ± 2% Klason lignin and 57 ± 4% crystalline cellulose, as determined by acetic-nitric digestion [31], and 10 ± 1% hemicellulose (Fig. 1a). Hemicellulosic monosaccharides were mostly xylose and glucose, with smaller amounts of galactose, arabinose, and mannose (Fig. 1b). Wood particles from WT poplar yielded about 33% G-lignin units and 67% S-lignin units, based on derivatization followed by reductive cleavage (DFRC) analysis (Table 1). By comparison, the high-S lines yielded 86 and 93% S subunits, while the high-G and high-5-OH-G lines yielded 54 and 57% and 18 and 42% S subunits, respectively. We next determined the distribution of reduced monolignols in soluble liquors resulting from CDL treatment by NMR spectroscopy. Two-dimensional ^1^H–^13^C heteronuclear single quantum coherence (HSQC) NMR gave estimates of the proportions of G and S units extracted into the liquors that trended with those from DFRC of wood particles (Table 1). Finally, as neither the DFRC method nor NMR spectroscopy was able to distinguish 5-OH-G from G units, we employed electrospray ionization (ESI) mass spectrometry (MS) to determine proportions of four reduced monolignol derivatives, H, G, 5-OH-G, and S units in the CDL liquors (Table 1). Most of the aromatic species were monomeric, but homo- and hetero-dimers and trimers were also detected, and when G- and 5-OH-G were summed, the proportions of each unit were in general agreement with both the DFRC and NMR results. We found WT to be about 8% 5-OH-G and 25% G, with slight reduction of 5-OH-G in the high-S lines. By contrast, RNAi suppression of *F5H* resulted in increase in the proportion of 5-OH-G, to 11 or 12%; RNAi suppression of endogenous *COMT* increased the proportion of 5-OH-G further to between 32 and 41%, depending on poplar line, at the expense of S units, which in one line was only 21% (Table 1). The small amounts of H-lignin detected were similar in WT and transgenic poplar lines.

**Table 1:** Lignin composition of poplar transgenic milled wood particles and products of catalytic delignification

Genotype	Lignin composition^a^											
	H			G^b^			OH-G^b^			S		
	DFRC (mol%)	NMR (mol%)	ESI (mol%)	DFRC (mol%)	NMR (mol%)	ESI (mol%)	DFRC (mol%)	NMR (mol%)	ESI (mol%)	DFRC (mol%)	NMR (mol%)	ESI (mol%)
Control (INRA 717) (1)	0.7 ± 0.0	nd	1.7 ± 0.0	32.7 ± 0.0	29	25.2 ± 1.5	–	–	8.2 ± 0.2	66.6 ± 0.0	71	65.0 ± 1.3
AtC4H:F5H-37 (2)	0.6 ± 0.1	nd	0.8 ± 0.0	6.3 ± 0.4	6	19.2 ± 1.5	–	–	6.0 ± 0.2	93.1 ± 0.3	94	73.9 ± 1.4
AtC4H:F5H-64 (3)	0.6 ± 0.1	nd	1.0 ± 0.0	13.7 ± 0.3	13	21.2 ± 0.7	–	–	6.1 ± 0.2	85.7 ± 0.4	87	71.6 ± 0.5
AtC4H:F5H2 RNAi (4)	0.3 ± 0.5	nd	2.1 ± 0.1	47.2 ± 0.6	42	33.2 ± 1.0	–	–	11.2 ± 0.5	52.4 ± 1.0	58	53.5 ± 1.4
35S:F5H2 RNAi (5)	0.9 ± 0.2	nd	2.4 ± 0.1	42.2 ± 1.0	36	28.6 ± 0.3	–	–	12.2 ± 0.2	56.8 ± 12.0	64	56.8 ± 0.4
AtC4H:COMTa RNAi (6)	0.8 ± 0.0	nd	1.7 ± 0.1	44.8 ± 0.3	37	24.0 ± 1.1	–	–	32.2 ± 1.1	54.4 ± 0.3	63	42.0 ± 2.3
35S:COMTa RNAi (7)	0.0 ± 0.0	nd	1.6 ± 0.1	79.2 ± 0.6	64	39.1 ± 1.8	–	–	41.0 ± 3.5	20.8 ± 0.6	36	18.3 ± 1.7

Composition in wood particles was determined by derivatization followed by reductive cleavage (DFRC); composition of reduced lignin units from catalytic conversion was determined by 2-D, ^1^H, ^13^C heteronuclear single quantum coherence (HSQC) NMR, and by electrospray ionization MS. Values from DFRC and ESI are the mean ± SD of three samples

nd, not detected; –, not determined

^a^DFRC was performed on milled wood particles; NMR and ESI were performed on the soluble liquors from catalytic delignification and reduction

^b^Neither DFRC nor NMR can distinguish G and OH-G units; total G represents the sum of these units

### Solubilization and gelatinization of cellulose and poplar cell walls in trifluoroacetic acid

**Fig. 2:**
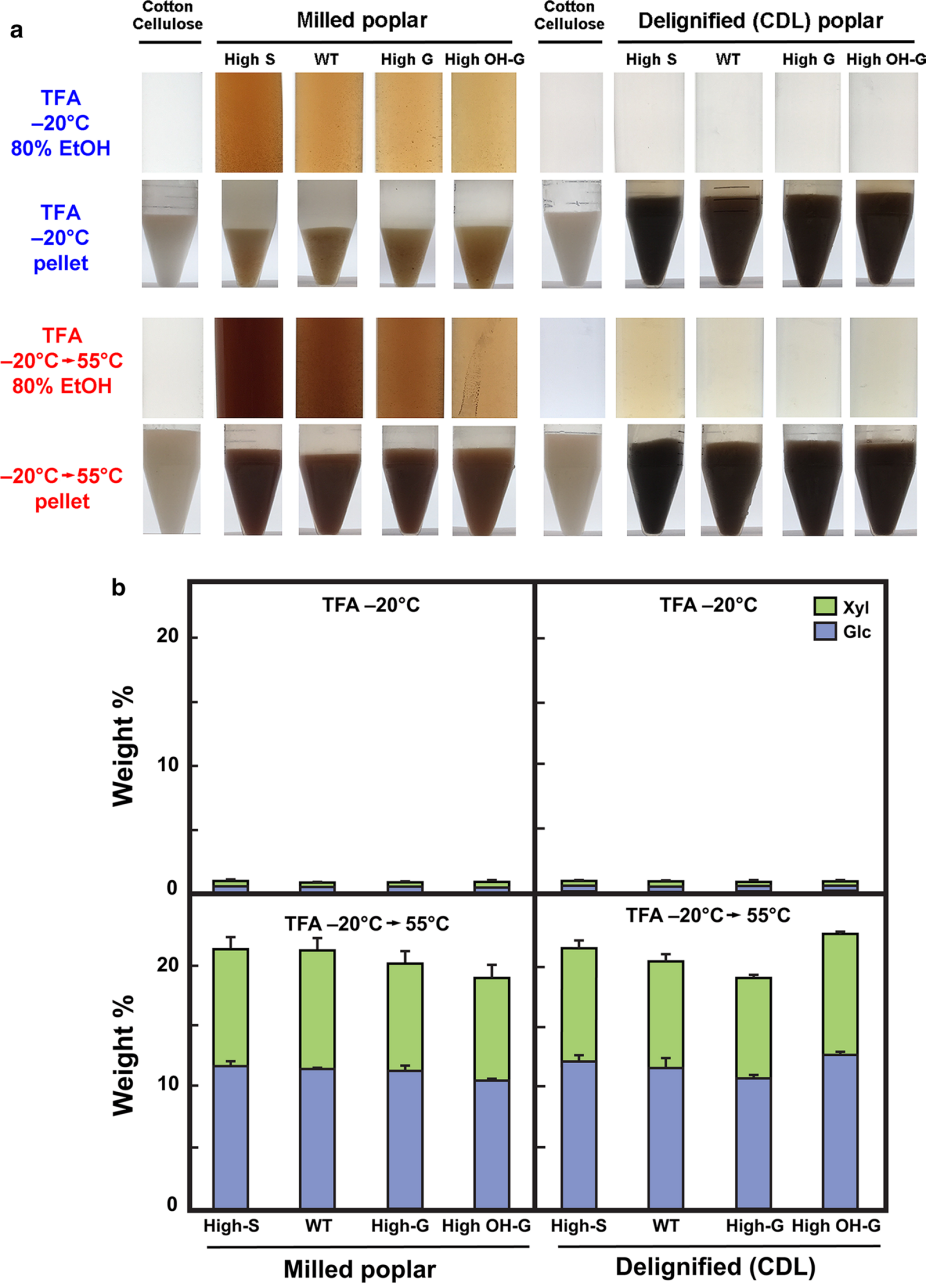
Comparison of low-temperature swelling and subsequent gelatinization behavior of transgenic variants of poplar with and without catalytic delignification.** a** Cotton linter cellulose (control) and milled wood particles from WT poplar (*Populus tremula* × *P. alba* cv. INRA 717-1B4) and three transgenic variants representing high S-lignin (*pAtC4H:AtF5H1-35*), high G-lignin* (pAtC4H:PtF5H2* RNAi) and high OH-G-lignin (*p35S:PtCOMTa* RNAi) compositions were swollen in two volumes of ice-cold TFA and incubated at –20°C for 15 h. Some samples were subsequently heated to 55°C for 5 h to gelatinize the swollen cellulose. Absolute ethanol was added to 80% (v/v), and insoluble material pelleted by centrifugation. Upper panels: Color of the TFA-soluble extract in 80% ethanol (v/v). Lower panels: After washing in 80% (v/v) ethanol in water, and water alone, the insoluble materials were homogenized and settled in the bottom of 15-ml conical tubes.** b** Weight % of Xyl and Glu recovered in the soluble fractions from TFA-swollen and gelatinized materials. Values are the means ± S.D. of three determinations

For subsequent experiments, we selected WT (1), an pAtC4H:*F5H* overexpressing (high-S) line (2), an pAtC4H:*F5H* RNAi (high-G) line (4), and a p35S:*COMTa* RNAi (high-5-OH-G) line (7) (Additional file 1: Table S1). Cotton linter cellulose swells at sub-zero (°C) temperatures and completely dissolves in TFA upon gentle heating [22]. Under similar conditions, WT and all lignin variants failed to swell in TFA at −20 °C, but swelling was observed upon subsequent heating to 55 °C (Fig. 2a). Little sugar was solubilized into TFA following low-temperature swelling, but subsequent heating released mostly xylose and glucose from wood particles of all genotypes (Fig. 2b). Addition of ethanol precipitated the gelatinized materials. Considerable browning occurred in the ethanol-soluble fraction, and the extent of browning was roughly correlated with S content (Fig. 2a). Upon CDL, all poplar genotypes swelled in low-temperature TFA, and heating did not increase swelling volume. The CDL treatments remove up to 90% of the lignin [25, 26], and consistent with these observations, the browning that occurred in the TFA treatments of milled WT poplar was all but eliminated in most samples, regardless of lignin composition (Fig. 2a). Similar low yields of xylose and glucose were obtained in low-temperature swelling in TFA. With subsequent heating after CDL, yields of xylose and glucose varied between 18 and 22% of starting weight, similar to materials that were not CDL-treated, indicating that delignification had little impact on the carbohydrate composition of the residues (Fig. 2b).

### Qualitative and quantitative analysis of crystalline and amorphous cellulose

**Fig. 3:**
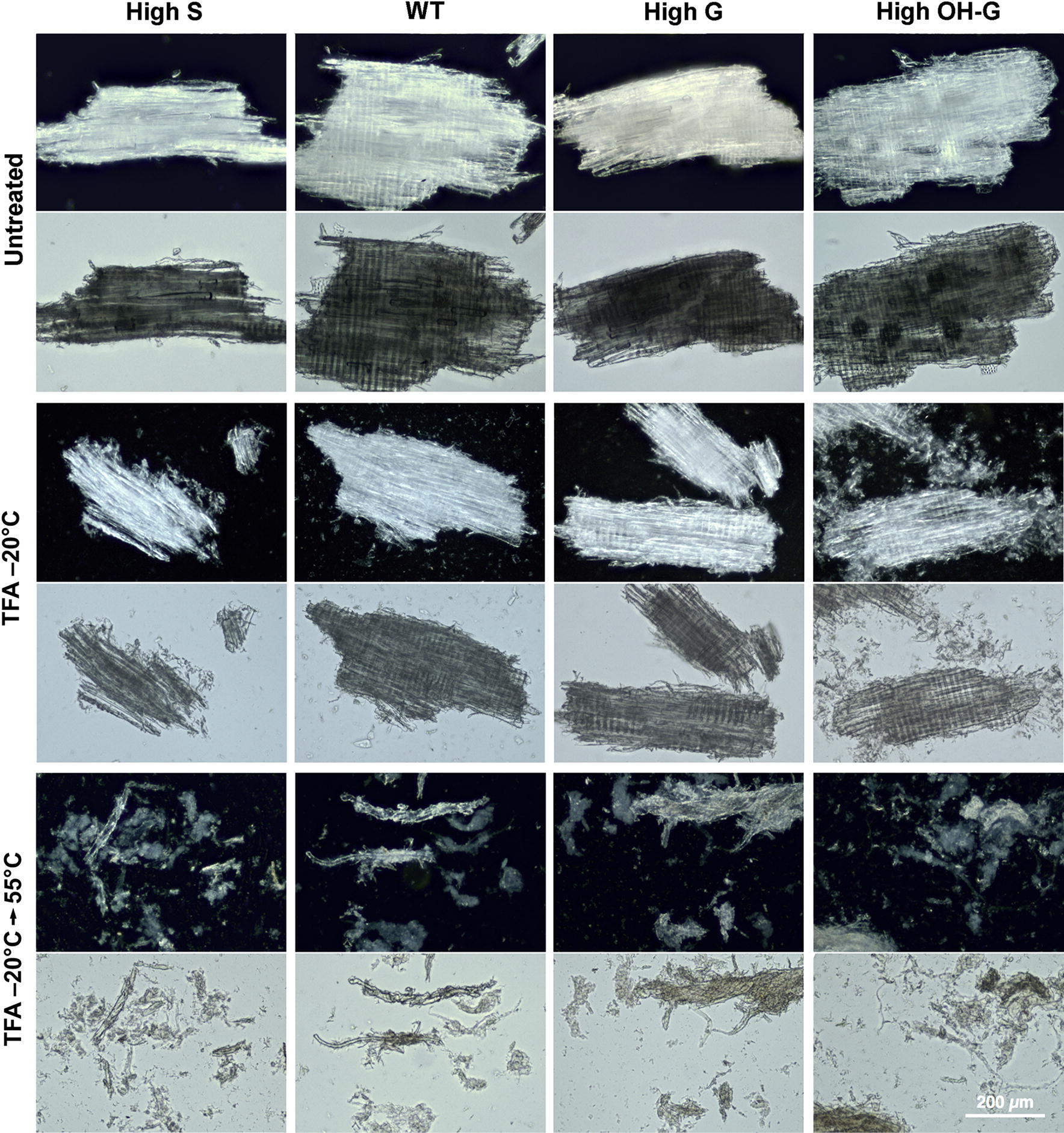
Reduced birefringence upon treatment of milled poplar wood particles after low-temperature swelling in TFA and subsequent gelatinization. Relative crystallinity of cellulose as determined by darkfield microscopy (upper panels) compared to differential interference contrast microscopy (lower panels) for each treatment. WT = (*Populus tremula* × *P. alba* cv. INRA 717-1B4), high S-lignin =* pAtC4H:AtF5H1*, high G-lignin =* pAtC4H:PtF5H2* RNAi, and high OH-G-lignin =* p35S:PtCOMTa* RNAi. Scale bar = 200 μm

**Fig. 4:**
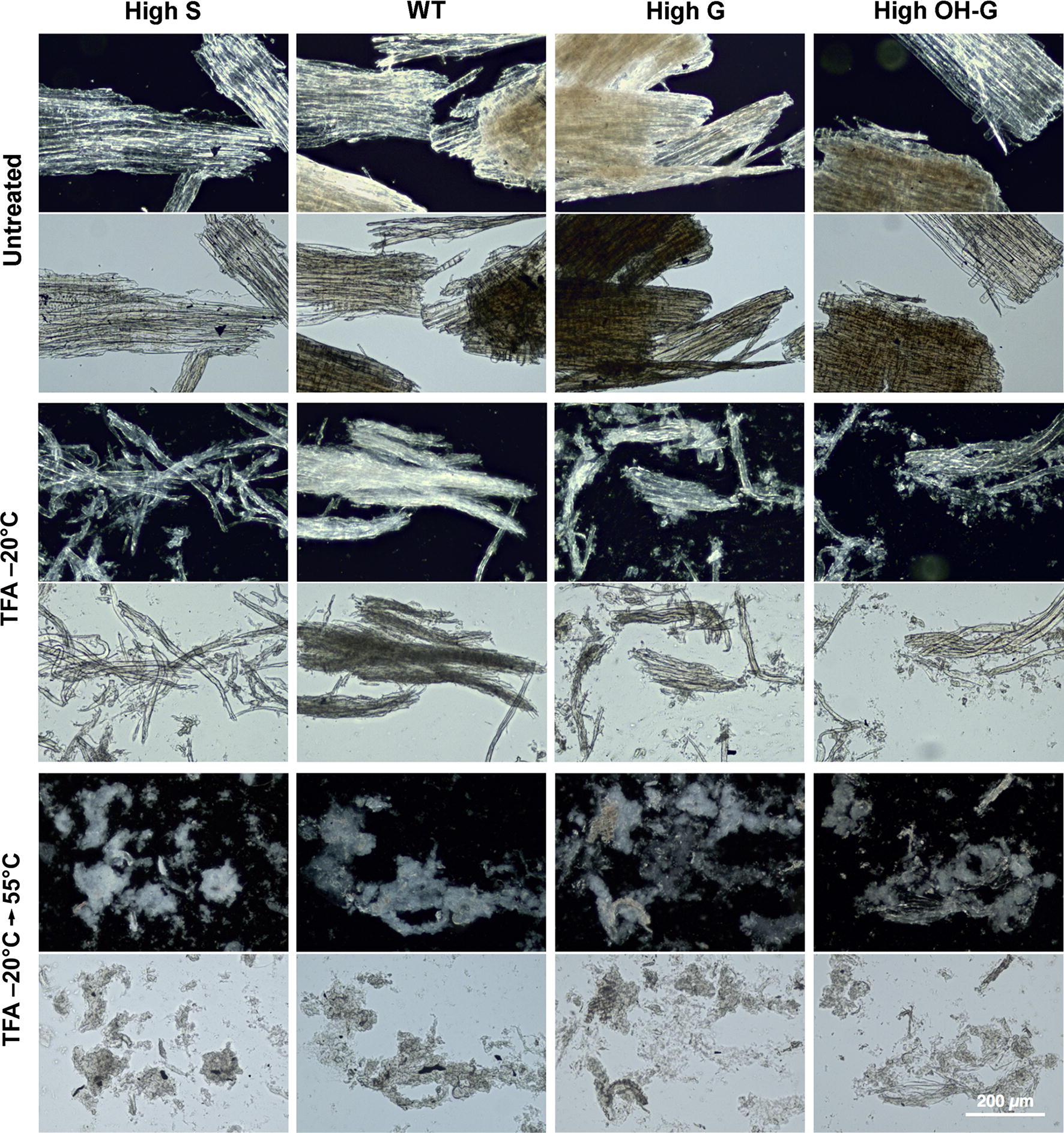
Reduced birefringence upon treatment of catalytically delignified (CDL) poplar wood particles after low-temperature swelling in TFA and subsequent gelatinization. Relative crystallinity of cellulose as determined by darkfield microscopy (upper panels) compared to differential interference contrast microscopy (lower panels) for each treatment. WT = (*Populus tremula* × *P. alba* cv. INRA 717-1B4), high S-lignin =* pAtC4H:AtF5H1*, high G-lignin =* pAtC4H:PtF5H2* RNAi, and high OH-G-lignin =* p35S:PtCOMTa* RNAi. Scale bar = 200 μm

Previously, we used dark-field microscopy to observe the reduced birefringence of crystalline cotton cellulose after low-temperature swelling [22]. By contrast, loss of birefringence was observed only after gelatinization of the milled poplar materials (Fig. 3). Upon heating, the particles fragmented and exhibited substantial cell separation, and birefringence was further reduced, regardless of lignin composition. Catalytic delignification of milled poplar wood particles of all genotypes resulted in substantial cell separation and reductions in birefringence upon low-temperature swelling alone in TFA (Fig. 4). Subsequent heating resulted in substantial loss of cell integrity and birefringence.

**Fig. 5:**
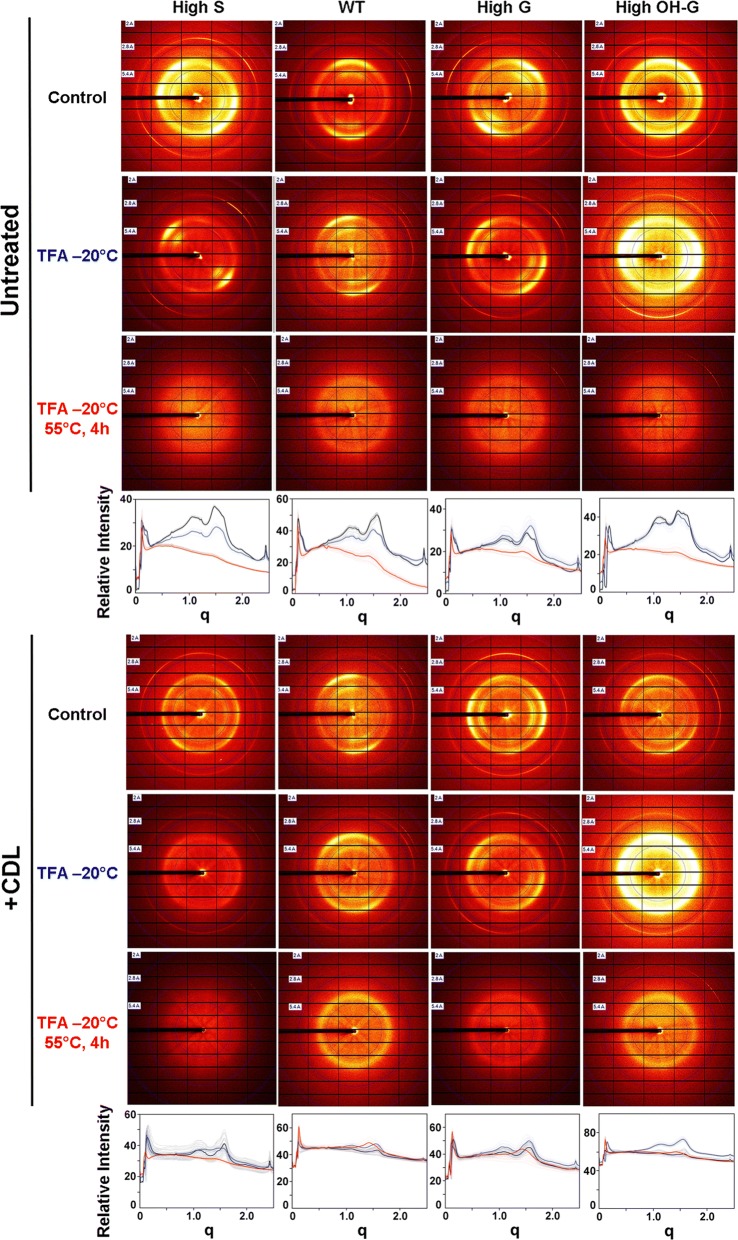
X-ray scattering of wild type and poplar variants with and without catalytic delignification (CDL). Upper panels: Control, untreated swollen in TFA at –20°C, and untreated swollen in TFA at –20°C, and heated to 55°C. Lower panels: Catalytically delignified (CDL) control, CDL material swollen in TFA at –20°C, and CDL material swollen in TFA at –20°C, and heated to 55°C. Plots of relative intensities are overlays of control (black), swollen (blue), and gelatinized (red) materials.WT = (*Populus tremula* × *P. alba* cv. INRA 717-1B4), high S-lignin =* pAtC4H:AtF5H1*, high G-lignin =* pAtC4H:PtF5H2* RNAi, and high OH-G-lignin =* p35S:PtCOMTa* RNAi.

The degree of loss of crystallinity in the poplar genetic variants after cold-swelling and heat-gelatinization in TFA was also estimated by X-ray diffraction, which was characterized by diminution or loss of sharp scattering peaks of cellulose Iα. The untreated WT control exhibited strong equatorial scattering and sharp meridionals, as expected for material with a high cellulose I content (Fig. 5). Modification of lignin composition did not significantly alter the overall organization of the cellulose microfibrils. Additional treatments led to weakening or loss of the prototypical equatorial and meridional reflections. Crystallinity was maintained to a large extent in poplar, regardless of lignin composition, and heat-induced swelling was required for its complete loss (Fig. 5a–c). Crystallinity of cellulose was not disrupted by CDL treatment of wood particles, but was diminished substantially upon cold-temperature swelling in TFA, particularly in the high-S lines. Crystallinity of the wood particles was considerably lowered but not eliminated upon heating (Fig. 5d–f). All CDL-treated materials exhibited greater intensity of background scattering, indicating an increase in disordered surface glucan chains.

### Resistance to acid hydrolysis

**Fig. 6:**
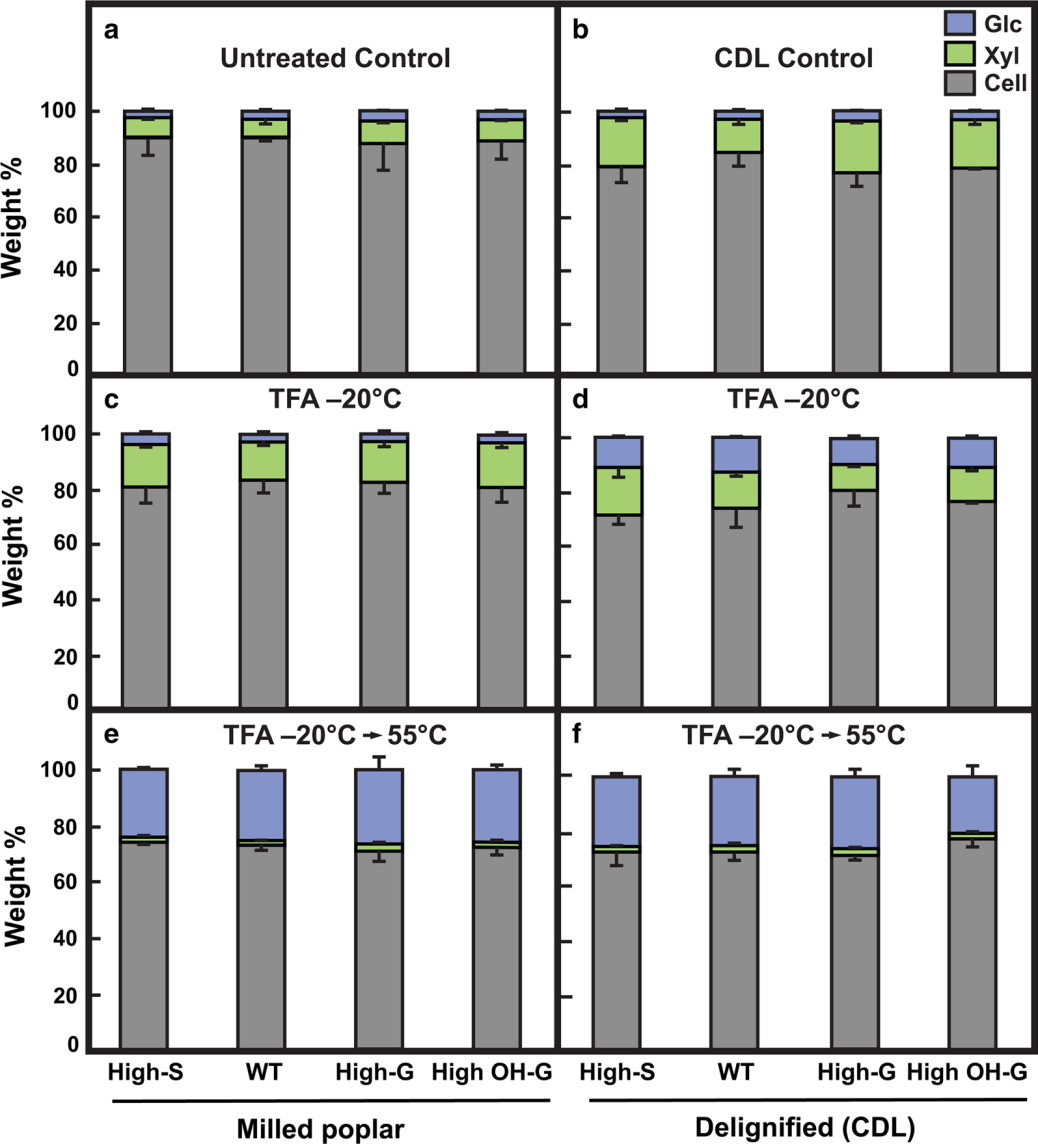
Proportions of sugar yields and resistant material from poplar variants with or without catalytic delignification to acid hydrolysis after swelling and gelatinization in TFA. Freeze-dried materials of untreated (**a**) and catalytically delignified (CDL) (**b**), low-temperature swollen in TFA without (**c**) and with CDL treatment (**d**), and TFA-swollen gels heated to 55°C for 5h without (**e**) or with CDL treatment (**f**) were hydrolyzed in 2 M TFA at 120°C for 90 min. Cellulose was determined in insoluble residue (in gray), and monosaccharides, glucose (in blue) and xylose (in green), were determined after conversion to alditol acetate derivatives and separation by GC-MS. WT = (*Populus tremula* × *P. alba* cv. INRA 717-1B4), high S-lignin =* pAtC4H:AtF5H1*, high G-lignin =* pAtC4H:PtF5H2* RNAi, and high OH-G-lignin =* p35S:PtCOMTa* RNAi. Values are the means ± S.D. of three determinations of weight % of carbohydrate in the insoluble material remaining after each extraction

Crystalline cellulose is resistant to hydrolysis in 2 M TFA at 120 °C for 90 min, a treatment that hydrolyzes all non-cellulose sugars for composition analysis. In particles of milled poplar wood, the amount of cellulose resistant to 2 M TFA was similar for all genotypes, regardless of lignin composition (Fig. 6a). Monosaccharide yields from low-temperature TFA-treated wood particles were only slightly increased over untreated controls, but a substantial proportion of xylose was removed from each genotype (Fig. 6c). However, after heating at 55 °C for 5 h in 2 M TFA the remaining xylan was hydrolyzed and about 22% of glucose from cellulose, regardless of genotype (Fig. 6e). Removal of lignin by CDL increased substantially the yield of xylose from all genotypes relative to untreated samples (Fig. 6a, b). When combined with low-temperature swelling, yields of glucose from cellulose were markedly increased in CDL-treated materials over controls (Fig. 6c, d). However, the yields of glucose were not improved over controls without CDL following 55 °C heat treatment in TFA (Fig. 6e, f).

### Enzymatic digestion of poplar biomass is enhanced by both TFA and CDL treatment

**Fig. 7:**
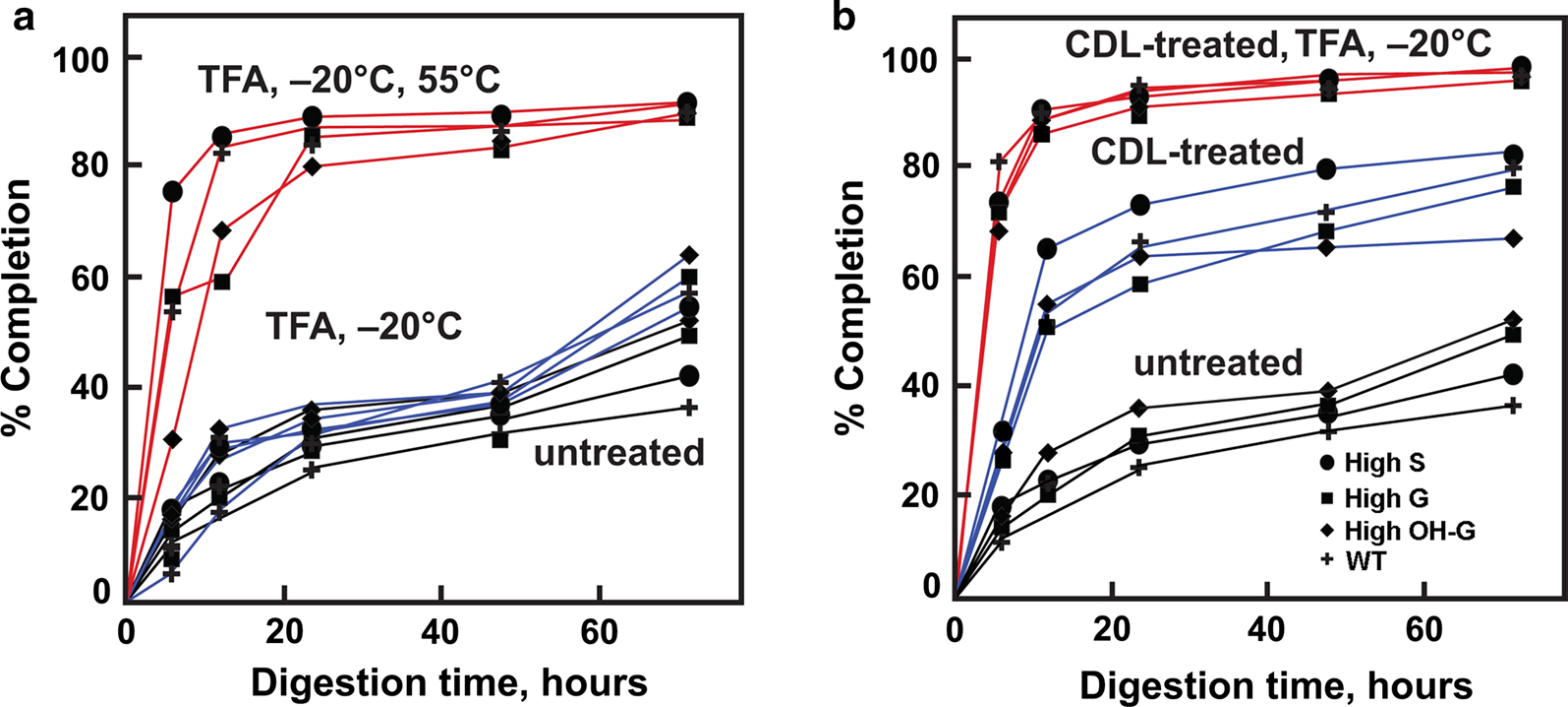
Rates of enzymatic hydrolysis of milled wood particles from wild-type (WT) and transgenic poplar lines from materials treated with TFA with or without catalytic delignification (CDL).** a** Progress of enzymatic hydrolysis of material untreated (black lines), swollen in TFA at –20 °C (blue lines), or gelatinized in TFA at 55°C (red lines).** b** Progress of enzymatic hydrolysis in untreated materials (black lines), CDL-treatment materials (blue lines), or CDL-treated materials swollen in TFA at –20 °C (red lines). WT = (*Populus tremula* × *P. alba* cv. INRA 717-1B4), high S-lignin =* pAtC4H:AtF5H1*, high G-lignin =* pAtC4H:PtF5H2* RNAi, and high OH-G-lignin =* p35S:PtCOMTa* RNAi. Enzyme hydrolysis was initiated by addition of 1 μL (0.09 FPU) of Cellic® Ctec2 (Novozymes) to 5 mg of milled particle preparations in 2 mL of 50 mM sodium citrate, pH 5.0, and incubated at 50°C for up to 72 h. Values are the means ± S.D. of three determinations.

Using a standard Ctec2 enzyme cocktail for saccharification assays, rates of enzymatic hydrolysis of low-temperature TFA-treated wood particles were only slightly increased over untreated controls for all genotypes (Fig. 7a). We have shown previously that tenfold higher amounts of enzyme loading improve final yield, but the substantial recalcitrance to digestion that remains is minimized by TFA [22]. However, after heating at 55 °C for 5 h, initial rates of enzymatic hydrolyses were significantly enhanced in the high-S poplar material, in which nearly 80% of theoretical yield was hydrolyzed after 6 h of digestion, compared to slightly above 50% for the WT and high-G lines, and only 30% for the high-5-OH-G line (Fig. 7a). Incubation beyond 40 h resulted in almost complete digestion, regardless of lignin composition. The CDL treatment of poplar material greatly enhanced the rates of enzymatic hydrolysis (Fig. 7b). Higher initial rates of hydrolysis were observed in the high-S line at 6 h and 12 h, even though almost all the lignin is removed by CDL treatment [25, 26]. Low-temperature TFA treatment alone of CDL-treated material resulted in greater than 80% completion in 12 h for all genotypes (Fig. 7b).

### Comparison of performance of poplar biomass lignin variants in chemical catalytic conversion of cellulose to biofuel substrates

**Fig. 8:**
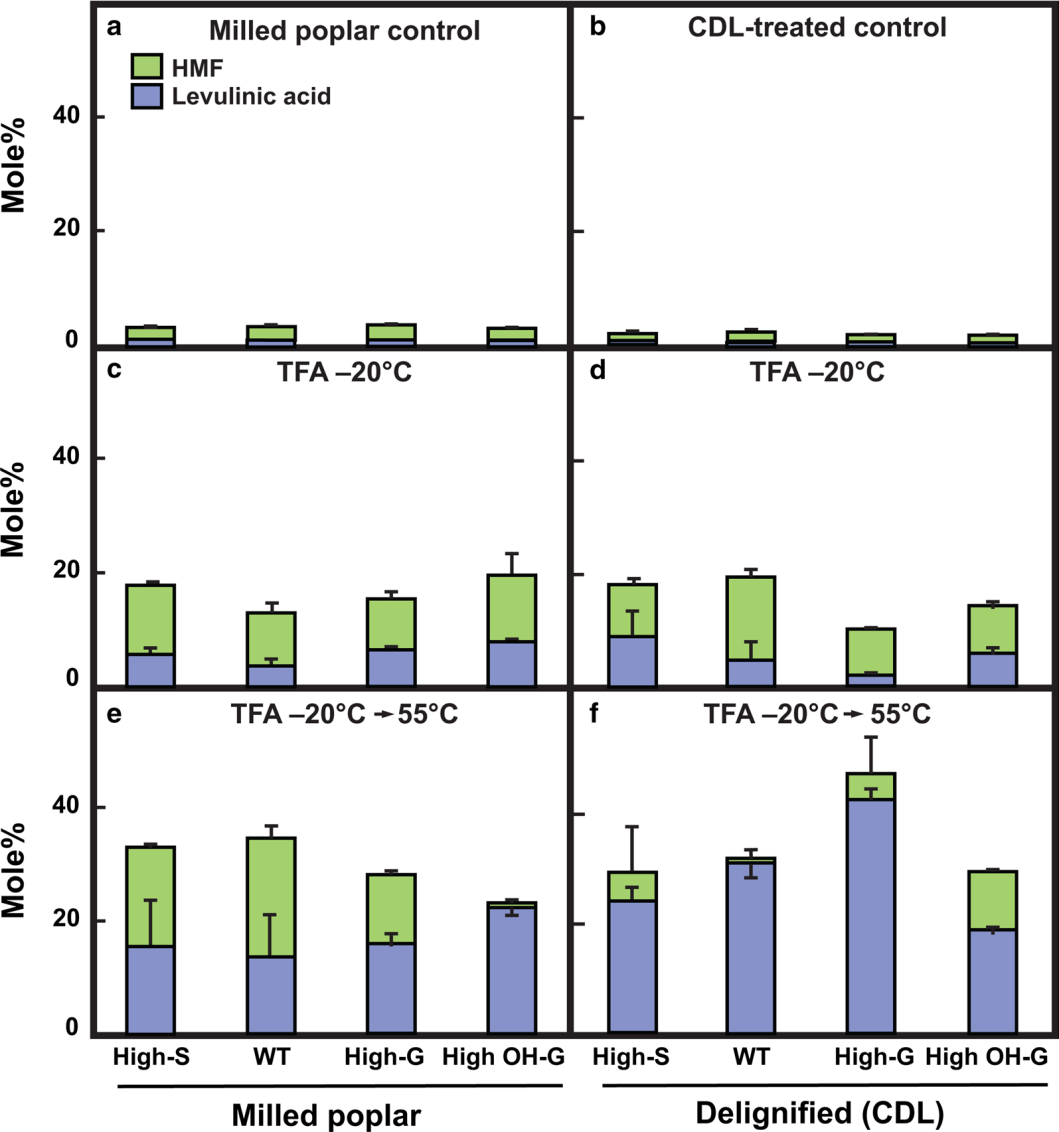
Molar conversion yields of HMF and levulinic acid from insoluble material untreated, swollen in TFA, and gelatinized in TFA for wild-type (WT) and transgenic poplar lines with and without catalytic delignification (CDL).** a** Milled poplar particles.** b** CDL-treated poplar particles.** c** Milled particles swollen in TFA at –20°C for 15 h.** d** CDL-treated particles swollen in TFA at –20°C for 15 h.** e** Milled particles swollen in TFA at –20°C for 15 h, then heated to 55°C for 4 h.** f** CDL-treatment particles swollen in TFA at –20°C for 15 h, then heated to 55°C for 4 h. WT = (*Populus tremula* × *P. alba* cv. INRA 717-1B4), high S-lignin =* pAtC4H:AtF5H1*, high G-lignin =* pAtC4H:PtF5H2* RNAi, and high OH-G-lignin =* p35S:PtCOMTa* RNAi. Monolignol distributions are provided in Table 1. Control particles and never-dried TFA-treated materials (50 or 75 mg) were suspended in 2 ml of 100 mM each of maleic acid and AlCl_3_ and reacted at 180 °C for 15 minutes. Values are the means ± S.D. of three determinations.

For untreated and CDL-treated poplar samples, the yields of HMF and LA from maleic acid and AlCl_3_ catalytic conversion of cellulose were low regardless of genotype (Fig. 8a, b). Despite the lack of swelling of control materials in cold TFA, yields of both biofuel substrates from lignified poplar particles were enhanced over fourfold by the low-temperature TFA treatment (Fig. 8c). Although low-temperature swelling in TFA was enhanced by CDL treatment, yields of HMF or LA were not enhanced further in the delignified material (Fig. 8d). However, yields of both products were increased further upon gelatinization of the TFA-swollen control poplar materials at 55 °C, with larger increases in yields of LA and more variable amounts of HMF recovery, depending on poplar genotype (Fig. 8e). Larger increases in proportion and amount of LA were observed in gelatinized material after delignification (Fig. 8f).

## Discussion

Low-temperature swelling in TFA substantially reduces recalcitrance of crystalline cellulose to both enzymatic digestion and chemical catalytic conversion. We proposed previously that, in a closed system to prevent loss of TFA, the relative ease of its recovery and regeneration by distillation makes it a potentially useful reagent for large-scale deconstruction of biomass and catalytic conversion to biofuel components and useful bioproducts [22]. In this study, we demonstrated that intact woody particles with a wide range of lignin compositions could be catalytically delignified and then residual cellulose is converted to desired products in either biological or chemical conversion pathways using a single-step swelling in cold TFA. Cellulose microfibrils remained refractory to enzymatic digestion in the absence of lignin, illustrating their intrinsic recalcitrance. However, the recalcitrance of crystalline cellulose to enzymatic digestion is eliminated to a large extent by cold-temperature swelling of the delignified material in TFA (Fig. 7b).

The concept of a second- or third-generation biorefinery is dependent on optimizing recovery of cellulose and other carbohydrates for their conversion to ethanol or butanol [32, 33], or catalytic conversion to liquid hydrocarbons [34–36]. For these end uses, biomass recalcitrance is generally considered to be the resistance of the carbohydrates in plant cell walls to microbial or enzymatic deconstruction [1, 2], the molecular basis of which is interaction between lignin and cellulose, which limits access by hydrolytic enzymes [37]. Although a small percentage of lignin residues has been converted into commercial products, such as surfactants, dispersants, or stabilizers [38, 39], a majority has been considered waste or burned to generate electricity [40]. For this reason, several technologies have been proposed to realize the vision of a lignin-first biorefinery that includes valorization of lignin-derived materials into high-value products [41–45]. The use of carbon nanoparticles supporting Zn-Pd or Ni to catalyze the removal of all detectable lignin is a breakthrough technology for a ‘lignin-first’ biorefinery strategy, where cellulose and other carbohydrates are left intact for subsequent downstream recovery and conversion to fuel or value-added molecules [25–27].

Lignin variants rich in native and novel aromatic subunits have been developed by genetic modification. Of several genetic approaches, one of the most promising is the enhanced expression of an *F5H* gene in poplar to produce lignin that is almost entirely composed of S units [46]. High-S-lignin wood substantially enhances Kraft pulping efficiency [47], and the same modification in Arabidopsis increases glucose yield by enzymatic digestion [48]. Overexpression of an *F5H* coupled with downregulation of *COMT* resulted in a unique form of lignin severely deficient in S and inferred to be rich in 5-OH-G subunits [49]. The significance of these and other genetic variants for the biorefinery is mitigation of energy costs involved in separations of different aromatic products; their substrates are isolated from different biomass sources rather than combined in a mixture derived from WT wood [27].

As CDL technologies eliminate the contribution of lignin to recalcitrance, we broadened this term to include features of biomass that disproportionately increase energy requirements, increase the cost and complexity of biorefinery operations, and/or reduce the recovery of biomass carbon into desired products [50]. Hence, factors influencing recalcitrance are not restricted to lignin–cellulose interactions at the atomic scale, but can involve a broad range of factors that vary among biomass types, from microscale cell–cell interactions to macroscale tissue organization that impacts comminution [50, 51]. For example, fiber and tracheary elements of woody tissues are tightly fused in the compound middle lamellae, comprising lignin and non-cellulosic polysaccharides [52, 53]. Treatments that oxidize lignin cross-links and extract or digest the non-cellulosic polysaccharides induce cell separation that increases saccharification yields in milled poplar material, and enhances cell separation in high-S lines (Yang et al. in review). Maleic acid-catalyzed depolymerization of xylans also results in dissolution of the compound middle lamellae in high-S Arabidopsis variant lines but not in high-G lines [54]. Similarly, CDL-treated materials showed both loss of crystallinity and increased cell separation, particularly in the high-S lines (Figs. 4, 6). The CDL markedly enhanced the rates of digestion of cellulose and xylan in saccharification assays (Fig. 7b). Therefore, the use of genetic variants is desirable for reducing sources of non-lignin-based recalcitrance.

## Conclusions

The ‘no carbon left behind’ principle of the lignin-first biorefinery is a two-stage process, where catalytic delignification to produce a value stream of aromatic products is followed by dissolution of cellulose microfibrils into more easily convertible glucan chains. Yields of xylose to furfural from poplar at 70% [28], and the TFA-enhancement of yields of HMF and LA from cellulose of over 40%, add to the aromatic products from CDL technology. As we gain understanding of the molecular bases of non-lignin recalcitrance, control of microfibril size and structure, microfibril bundling, and cell–cell adhesion become feasible targets of genetic modification. Cellulosic genetic variants might work synergistically with downstream TFA treatment, as we have demonstrated that lignin variants do for CDL treatments. With greater than 50% of the lignin from CDL recovered in reduced monolignols [25, 26], and nearly 100% of the xylose and glucose digested enzymatically in 6 h after swelling in TFA, we have made significant progress toward the ‘no carbon left behind’ principle of the lignin-first biorefinery.

## Methods

### Generation of lignin genetic variants in poplar

For overexpression, cDNA of the Arabidopsis *F5H1* gene (At4g36220) was first cloned into a Gateway-compatible entry vector and then transferred to plant binary vectors pCC0995, an overexpression destination vector with the CaMV *35S* promoter, and pCC0996, an overexpression destination vector in which the candidate gene is driven by the promoter from the Arabidopsis *C4H* gene (*AtC4H*) [55].

For downregulation, RNAi constructs were designed from conserved consensus sequences of poplar *F5H2* (*P. trichocarpa* ‘Nisqually’ 1/383-2499) and *COMTa* (*P. trichocarpa* × *P. deltoides* H11-11) genes were cloned into a Gateway-compatible binary vector in the forward and reverse directions to facilitate hairpin formation, giving rise to pCC0989, an RNAi destination vector with the *35S* promoter, and pCC0994, an RNAi destination vector with *AtC4H* promoter. We have previously shown that the *C4H* promoter is superior to the *35S* promoter for targeting lignifying cells and for modifying lignin monomer composition [56, 57], so we expected the *C4H*-driven constructs to be more efficacious. However, high levels of *C4H* suppression or *C3′H* expression may lead to extreme and deleterious phenotypes, so we generated constructs containing the *35S* promoter in parallel, in an attempt to generate transformants with a broader spectrum of phenotypes. The *AtF5H* overexpression, *PtCOMT1* RNAi, and *PtF5H* RNAi constructs are listed in Additional file 1: Table S1.

The parental WT hybrid poplar clone for all lignin variant lines was INRA 717-1B4. Methods for the production of transgenic lines have been described previously [55, 56]. Briefly, cells in leaf discs from in vitro-cultured shoot tips were transformed with *Agrobacterium tumefaciens* and used to regenerate whole plants using direct shoot organogenesis to reduce or eliminate somaclonal variation [58, 59]. Shoots were rooted on a selection medium, acclimated in the laboratory, and grown in a greenhouse before being transplanted in the field. For all lines, T-DNA insertion was confirmed by the polymerase chain reaction (PCR) using transgene-specific primers. Expression of transgenes was verified via quantitative real-time PCR (ABI Prism 7000 Sequence Detection System; Applied Biosystems) according to the manufacturer’s protocol.

### Plantation establishment and maintenance

The field site was sprayed with Roundup^®^ (Monsanto; St. Louis, MO), at the rate specified on the label, in 1.2-m strips, which were tilled following plant death. The bare-soil rows were then treated with the pre-emergent herbicides Pendulum^®^ and Aquacap^®^ (Monsanto), at rates specified on the labels. Row centers were 3 m apart, and planting positions within rows were flagged at ~ 2-m intervals. Trickle irrigation line (T-tape) was anchored down the center of each plant-row. A 2.3 m tall plastic mesh deer fence (Deer Busters fencing, 650-lb breaking strength) was installed around the perimeter of test site. As needed, the alleys between plant-rows were mowed, and weeds within the plant-rows were either hand-sprayed with a backpack sprayer or dome-sprayed by tractor with Roundup^®^ and Transline^®^ (Monsanto).

Planting holes (20-cm diameter) for each tree were bored with a power auger. Trees were hand-watered promptly after planting and irrigated as needed thereafter. Planting operations were conducted in June and October 2008, May 2009, and June 2010. In May 2010, all trees that had been planted up to that point were coppiced to near ground level to eliminate the potential for seed production, rejuvenate the population, and provide material for analysis and vegetative propagation [60]. Trees were coppiced again in April 2011 and in March 2014. Within a month of each coppicing, trees were “singled” by removing all stump sprouts except the most dominant leader, and root suckers [61].

### Tree harvest and processing

In March 2014, coppiced field trees (~ 10 cm in diameter at 1.5-m height) were sawn into 30-cm lengths in the field and stored in open milk crates in a walk-in freezer at − 4 °C for 1 to 3 months. Multiple ramets of both control and transgenic lines were bulked by line. Thus, individual trees within a given line and different sampling positions (including main stems and larger branches) provided a single, representative sample for that line. Between April and June 2014, the stems were removed from the freezer, oven dried at 45 °C for 3 to 7 days, and subsequently stored in a lab to await processing. Bark was manually peeled from the oven-dried stems using a spoke shave, and the debarked stems were subsequently knife-milled to pass through a ¼″ screen by Hazen Research (Golden, CO). Knife-milled poplar wood was milled further to pass through a 20-mesh (1 mm) screen with a Wiley Mill (Thomas Wiley; Swedesboro, NJ, USA).

### Treatment with TFA

Depending on experiment, 50- or 100-mg samples of cotton linter cellulose (Sigmacell; product No. S5504T) and milled wood particles from WT and transgenic poplars were suspended in 1:20 (w/v) ratios of ice-cold 99% TFA (Sigma-Aldrich) in 15-mL glass centrifuge tubes sealed with a Teflon^®^-lined screw caps, and incubated at − 20 °C for 15 h. After incubation, samples were either vortexed with five volumes of absolute ethanol (0 h) or incubated for 5 h at 55 °C before vortex mixing in the ethanol solution. Control samples in water were mixed with five volumes of ethanol. The insoluble solids and gels centrifuged at 1200×*g* in a swinging-bucket rotor for 5 min, and the pellets were washed four times with 12 mL of 80% ethanol in water (v/v), followed by four rinses with water. For lignocellulosic poplar materials, absorbance of the brown solutions was determined between 400 and 700 nm and correlated to S-lignin composition. Duplicate 2-mL samples of the TFA-ethanol solution were saved, and 0.5 mL of *tert*-butyl alcohol was added before the mixtures were dried under a stream of N_2_ at 40 °C in 4-mL glass tubes. The particulates were suspended in water and stored at 4 °C for further analysis, or freeze dried. Values reported are the mean ± SD of three samples.

### Catalytic delignification

Catalytic delignification was carried out in triplicate essentially as described previously [26]. Briefly, 1.0 g of 20-mesh poplar biomass, 0.10–0.15 g of 10 wt% Ni/C loaded into 325-mesh microporous cage, and 45 mL methanol were added to a 75-mL stainless steel Parr reactor (Parr Instruments; Moline, IL, USA). The reactor was charged with 35 bar UHP-grade H_2_, (Matheson; Montgomeryville, PA, USA) heated to 225 °C, and the reaction mixture was stirred at 700 rpm and maintained at the reaction temperature for 12 h. The reaction was terminated by cooling the reactor to ambient temperature. The reaction mixture was filtered through 11-micron filter paper (Whatman; Sigma-Aldrich) to separate the liquid phase containing aromatic products from the solid residue containing mainly cellulose and hemicellulose. The insoluble residue was washed with additional methanol to remove any remaining phenolic products on the solid surface and air dried.

### Determination of cellulose and lignin composition

Crystalline cellulose content was determined by acetic-nitric digestion [31]. The DFRC was performed according to Lu and Ralph [62], as modified by Li et al. [63]. Briefly, milled poplar particles were suspended in acetyl bromide/acetic acid solution (20:80, v/v) with 4,4′-ethylidenebisphenol as an internal standard. After evaporating the solvent under a stream of N_2_ gas, dioxane/acetic acid/water (5:4:1, v/v/v) and zinc dust were added to cleave the solubilized lignin. The reaction products were purified on a C-18 SPE column (Supelco) and acetylated with pyridine/acetic anhydride solution (2:3, v/v). The resulting lignin monomer derivatives were separated by gas–liquid chromatography on 30-m × 0.32-mm HP-5 capillary column (Agilent 19091J-413) using a temperature gradient of 140 °C to 240 °C at 3 °C/min, with a hold at 240 °C for 0.5 min, and then 240 to 310 °C at 30 °C/min, and a hold at 310 °C for 10 min. The monolignol derivatives were detected by flame-ionization using relative molar response factors of 0.80, 0.83, and 0.74 determined using H, G, and S standards. Values reported are the mean ± SD of three samples.

Lignin monomer composition was also determined on the dried products of CDL, which were dissolved in 0.8 mL CDCl_3_ (Sigma-Aldrich), before 2D ^1^H, ^13^C HSQC spectra were acquired on a Bruker 800 MHz spectrometer (http://www.bruker.com/) equipped with a *z*-gradient QCI cryoprobe at a sample temperature of 25 °C. 2D data acquisition times were 84 ms for ^1^H (direct dimension) and 4 ms for ^13^C (indirect dimension). Free induction decays (FIDs) for the ^1^H and ^13^C dimensions were processed using a sine-bell square window function, zero-filled, and Fourier-transformed. Baseline corrections were applied to the ^1^H dimension. Chemical shifts were referenced by the solvent peak (^1^H, 7.26 ppm; ^13^C, 77.0 ppm).

For electrospray ionization mass spectrometry, the dried products of CDL were dissolved in water and then diluted 1:1 with aqueous 40 mM sodium acetate. Mass analysis was obtained in positive mode with an Agilent 6545 Q-TOF mass spectrometer with ESI capillary voltage of + 3.5 kV, an N_2_ temperature of 320 °C, a drying gas flow rate of 8.0 mL/min, a nebulizer gas pressure of 35 psig, a fragmentor voltage of 135 V, a skimmer voltage of 65 V, and an octupole radio-frequency (OCT RF) voltage peak-to-peak (Vpp) of 750 V. Mass data (from *m/z* 80 to1100) were collected using Agilent MassHunter Acquisition software (v. B.06). MS/MS was performed in a data-dependent acquisition mode. Mass spectral data analysis used Agilent MassHunter Qualitative Analysis (v. B.07) software. Values reported are the mean ± SD of three samples.

### Dark-field and differential interference contrast microscopy

After low-temperature swelling and gelatinization, washed insoluble milled poplar materials were placed on glass microscope slides without additional treatment or staining. Images were captured using a Nikon C1 Plus microscope (Nikon; Tokyo, Japan) configured for either dark field or differential interference contrast (DIC) illumination and a SPOT RTKE CCD camera (Diagnostic Instruments; Sterling Heights, MI). FIJI (ImageJ) was used to rotate, crop, normalize brightness, and convert 16-bit color images to 8-bit grayscale images [22].

### X-ray diffraction

X-ray diffraction patterns of wood particles were collected using a 5-μm X-ray beam at GM/CA, beamline 23ID-B at the Advanced Photon Source at Argonne National Laboratory [64]. Exposure times were approximately 1 s with a sample-to-detector distance of 300 mm and X-ray wavelength of 1.033 Å. For each sample, 10–20 diffraction patterns were collected and circularly averaged about the center of the pattern and the resulting intensity curves averaged over all patterns collected. Intensities were plotted as a function of momentum transfer, *q*, where *q* = 4*π* sin(*θ*)/*λ*; *θ* is half the angle between incident and scattered beam and *λ* is the wavelength of the X-rays.

### Hydrolysis of lignocellulosic material

Samples (1 mg) of dry poplar cellulosic materials were suspended in 1 mL of 2 M TFA containing 500 nmoles of *myo*-inositol (internal standard) in 4-mL glass conical vials sealed with Teflon^®^-lined screw caps. The samples were heated to 120 °C for 90 min with occasional shaking. After cooling, the remaining insoluble material was pelleted by centrifugation at 4000×*g* for 5 min. The clear supernatant liquid was transferred to a 4-mL glass vial and dried under a stream of N_2_ at 45 °C. The pellet was washed twice with water followed by centrifugation before being suspended in 0.8 mL of water. Glucose equivalents were determined by phenol–sulfuric acid assay [65].

The dried soluble fraction was hydrolyzed in 1 mL of 2 M TFA at 120 °C for 90 min, then 0.5 mL of *tert*-butyl alcohol was added and the samples were mixed before being dried under a stream of N_2_ at 45 °C. The dried hydrolysates were reduced with NaBH_4_ and imidazole-catalyzed acetylation as described previously [66]. Alditol acetates of the monosaccharides recovered were identified and quantified by GC–MS, with *myo*-inositol as the internal standard. Values reported are the mean ± SD of three samples.

### Enzymatic digestion of lignocellulosic materials

Enzymatic hydrolysis experiments were performed with 5 mg of poplar wood particles suspended in 2 mL of 50 mM sodium citrate, pH 5.0, with 1 µL of Cellic™ Ctec2 (Novozymes) to bring to 1.8 FPU/g material (corresponding to 1.5-μg protein/mg) in 4-mL glass centrifuge tubes sealed with a Teflon^®^-lined screw caps. Enzymatic hydrolysis was carried out at 50 °C in a thermostatically controlled rotary-hybridization oven. During hydrolysis, samples were taken at intervals, and ethanol added to 80% (v/v) to precipitate unextracted material, and the suspension was pelleted by centrifugation for 5 min at 12,000×*g*. The total sugar content in supernatant and pellet were determined as glucose equivalents using a phenol–sulfuric acid assay [65]. Values reported are the mean ± SD of three samples.

### Catalytic conversion of lignocellulosic materials

Samples of 50 or 75 mg of wood particles that were swollen under low temperature and heat-gelatinized were hydrolyzed to glucose and converted sequentially to LA and HMF, with formic acid being produced by a parallel reaction during LA production. Maleic acid and AlCl_3_ were used as catalysts at concentrations of 100 mM, according to the methods described by Zhang et al. [67, 68]. Briefly, the never-dried insoluble materials were assayed for total sugar and suspended in 100 mM each of maleic acid and AlCl_3_ in deionized water. This reaction suspension was placed in a 3.5-mL 316L stainless steel reactor tube (8 mm diameter, 2.1 mm wall thickness, 70 mm length) with 12-mm Swagelok tube end fittings (Swagelok Indiana; Indianapolis, IN, USA). The reactor tubes were heated to 180 °C in a Tecam SBL-1 fluidized bath (Cole-Parmer; Vernon Hills, IL, USA), using a warm-up period of 2 min. Tubes were cooled to ambient temperature after 15 min by immersion in cool water. The LA and HMF concentrations were measured by high-pressure liquid chromatography (HPLC) using a Waters 1525 pump and a Waters 2412 refractive index detector (Waters; Milford, MA). Products were loaded on a 300-mm × 7.8-mm column of AMINEX HPX-87H (BioRad; Hercules, CA) and separated in a mobile phase of 5 mM H_2_SO_4_ with 5% (w/w) acetonitrile to facilitate separation of glucose and maleic acid. The column flow rate was 0.6 mL/min, and the column was maintained at 65 °C. All concentrations were determined by external calibration standards. Values reported are the mean ± SD of three samples.

## Additional file

**Additional file 1: Table S1.** Plasmid constructs used in overexpression of an Arabidopsis *F5H1* gene and RNAi-knockdowns of poplar (*Populus trichocarpa*) *F5H* and *COMTa* transcripts driven by a vascular-specific (*AtC4H*) or constitutive (CAMV*35*-*S*) promoters.

## Data Availability

All data generated or analyzed during this study are included in this published article and its additional file. Plant materials used in this study are available from corresponding author, Richard Meilan [E-mail: rmeilan@purdue.edu; Tel. 765-496-2287].

## Abbreviations

AFEX: ammonia fiber expansion
BMIM: 1-butyl-3-methylimidazolium
CaMV: cauliflower mosaic virus (*35S* promoter)
CDL: catalytic delignification
C4H: cinnamate 4-hydrolase
COMT: caffeic acid-*O*-methyltransferase
DIC: differential interference contrast
ESI-MS: electrospray ionization–mass spectrometry
F5H: ferulate 5-hydroxylase
G: guaiacyl
H: *p*-hydroxyphenyl
HMF: 5-hydroxymethylfurfural
HSQC: heteronuclear single quantum coherence
NMMO: *N*-methylmorpholine *N*-oxide
NMR: nuclear magnetic resonance
PCR: polymerase chain reaction
RNAi: RNA interference
S: syringyl
TFA: trifluoroacetic acid
