# Supplementary material for: Overcoming cellulose recalcitrance in woody biomass for the lignin-first biorefinery
Source: Biotechnol Biofuels. 2019 Jun 29;12:171. doi: 10.1186/s13068-019-1503-y (PMC6599248; doi:10.1186/s13068-019-1503-y)
Supplement: Supplementary file 1 — Additional file 1: Table S1. Plasmid constructs used in overexpression of an Arabidopsis F5H1 gene and RNAi-knockdowns of poplar (Populus trichocarpa) F5H and COMTa transcripts driven by a vascular-specific (AtC4H) or constitutive (CAMV35-S) promoters. [file 13068_2019_1503_MOESM1_ESM.docx]

**Additional file 1: Table S1.** Plasmid constructs used in over-expression of an Arabidopsis *F5H1* gene and RNAi-knockdowns of poplar (*Populus trichocarpa*) *F5H* and *COMTa* transcripts driven by a vascular-specific (*AtC4H*) or constitutive (CAMV*35-S*) promoters.

**______________________________________________________________________________**

**Name Promoter Gene or Target Gene (line#) Type**

pCC1063 AtC4H *AtF5H1-37* (2) Over-expression

pCC1063 AtC4H *AtF5H1-64* (3) Over-expression

pCC1035 AtC4H *PtF5H2* (4) RNAi-knockdown

pCC1020 CaMV-35S *PtF5H2* (5) RNAi-knockdown

pCC1036 AtC4H *PtCOMT1* (6) RNAi-knockdown

pCC0998 CaMV-35S *PtCOMT1* (7) RNAi-knockdown

**______________________________________________________________________________**
